# Elevated Adiponectin Levels Suppress Perivascular and Aortic Inflammation and Prevent AngII-induced Advanced Abdominal Aortic Aneurysms

**DOI:** 10.1038/srep31414

**Published:** 2016-09-23

**Authors:** Dick Wågsäter, Emina Vorkapic, Caroline M. W. van Stijn, Jason Kim, Aldons J. Lusis, Per Eriksson, Rajendra K. Tangirala

**Affiliations:** 1 Division of Drug research, Department of Medical and Health Sciences, Faculty of Health Science, Linköping University, Linköping, Sweden; 2 Division of Endocrinology, Diabetes and Hypertension, David Geffen School of Medicine, University of California, Los Angeles, USA; 3 Division of Cardiology, David Geffen School of Medicine, University of California, Los Angeles, USA; 4 Atherosclerosis Research Unit, Center for Molecular Medicine, Department of Medicine, Karolinska Institute, Stockholm, Sweden

## Abstract

Abdominal aortic aneurysm (AAA) is a degenerative disease characterized by aortic dilation and rupture leading to sudden death. Currently, no non-surgical treatments are available and novel therapeutic targets are needed to prevent AAA. We investigated whether increasing plasma levels of adiponectin (APN), a pleiotropic adipokine, provides therapeutic benefit to prevent AngII-induced advanced AAA in a well-established preclinical model. In the AngII-infused hyperlipidemic low-density lipoprotein receptor-deficient mouse (LDLR^−/−^) model, we induced plasma APN levels using a recombinant adenovirus expressing mouse APN (AdAPN) and as control, adenovirus expressing green florescent protein (AdGFP). APN expression produced sustained and significant elevation of total and high-molecular weight APN levels and enhanced APN localization in the artery wall. AngII infusion for 8 weeks induced advanced AAA development in AdGFP mice. Remarkably, APN inhibited the AAA development in AdAPN mice by suppressing aortic inflammatory cell infiltration, medial degeneration and elastin fragmentation. APN inhibited the angiotensin type-1 receptor (AT1R), inflammatory cytokine and mast cell protease expression, and induced lysyl oxidase (LOX) in the aortic wall, improved systemic cytokine profile and attenuated adipose inflammation. These studies strongly support APN therapeutic actions through multiple mechanisms inhibiting AngII-induced AAA and increasing plasma APN levels as a strategy to prevent advanced AAA.

AAA, a common aortic disease affecting up to 7% of men over 65 years, is associated with enhanced transmural inflammation, dilation and rupture of abdominal aorta. The key features of AAA pathogenesis include: infiltration of the aortic wall by inflammatory cells including macrophages, T-lymphocytes and mast cells, degeneration of vascular smooth muscle cells (VSMC) and degradation of extracellular matrix (ECM) components, collagen and elastin^1^,^2^. The major cellular infiltrates present in AAA are dominated by macrophages, T-lymphocytes, neutrophils, and mast cells, all of which produce a number of pro-inflammatory cytokines, chemokines and extracellular matrix proteases involved to the development and progression of AAA^3^,^4^. Previous studies have assigned the development of AAA to atherosclerosis^5^. Although both AAA and atherosclerosis share common risk factors including smoking, age, hyperlipidemia, hypertension and obesity^6^, the pathogenic mechanisms of AAA distinct from atherosclerosis remain poorly understood^4^. Obesity contributes to chronic inflammation due to altered adipose tissue secretion of adipokines which have has been linked to vascular function^7^. Although inflammatory adipokines, resistin and leptin and complete deficiency of anti-inflammatory adipokine APN have been linked to AAA^8^,^9^, their roles in the pathogenesis of AAA remain to be established^10^.

APN, a pleiotropic adipokine which exerts profound anti-inflammatory and atherogenic effects in vascular cells, is potential therapeutic target to prevent vascular inflammation and damage^11^,^12^. Circulating APN levels are reduced in obesity, the metabolic syndrome and cardiovascular disease^13^. The biological actions of APN are mediated through interaction with two major specific cell-surface receptors, AdipoR1 and AdipoR2 (AdipoRs) expressed in vascular cells, through activation of AMPK, PPAR-α and ceramide signaling pathways^14^,^15^. Macrophages play a key role in AngII-mediated vascular inflammation and AAA development^4^. Recently, we identified that the APN anti-inflammatory actions in macrophages are regulated by macrophage polarization and activation status^16^. In previous studies, we have established the protective role of APN against AngII-induced vascular inflammation and accelerated atherosclerosis in the hyperlipidemic LDLR^−/−^ model^11^. Consistently, a recent report showed that complete APN knockout in apolipoprotein E-deficient mice increased AngII-induced inflammation and early AAA^8^. In the present study, we investigated the therapeutic potential of increasing plasma APN levels to prevent advanced AAA in the well-established AngII-induced hyperlipidemic LDLR^−/−^ model in which AngII suppressed endogenous APN expression. We addressed the hypothesis that APN expression increasing plasma APN levels inhibits aortic, perivascular and visceral adipose inflammation, medial degeneration and elastin degradation in the aneurysmal wall to prevent advanced AAA development.

## Results

### Adiponectin expression and elevated plasma adiponectin oligomer levels in AngII-infused hyperlipidemic LDLR^−/−^ mice

**Figure 1:**
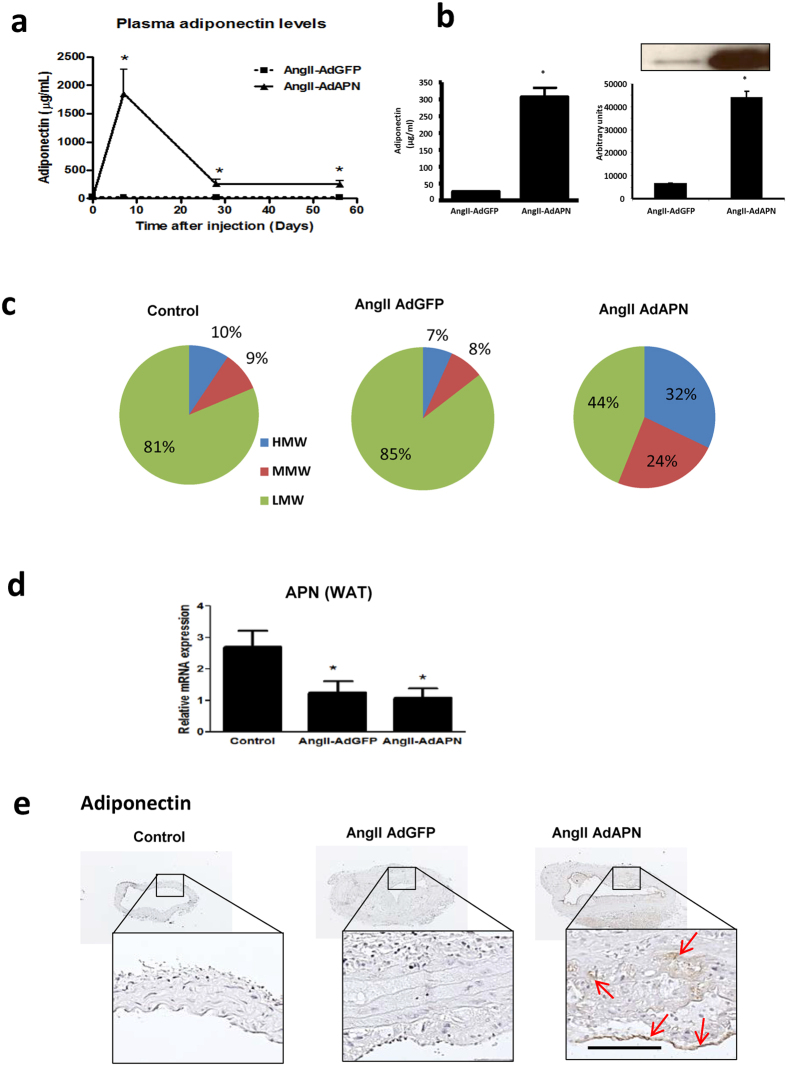
Adiponectin expression and plasma adiponectin levels in AngII-infused LDLR^−/−^ mice fed high-fat diet and injected AdAPN or AdGFP adenovirus during 8 weeks. (**a**) Plasma adiponectin levels at 8 weeks after injection measured by ELISA. (AdGFP n = 14 and AdAPN n = 10/group, *p < 0.001 Student’s t-test. (**b**) Western blot analysis of plasma APN levels at 8 weeks of adiponectin expression quantified by densitometry with NIH ImageJ software. (**c**) Distribution of circulating APN oligomeric forms quantified by gel electrophoresis, HMW, high molecular weight, MMW, medium molecular weight, LMW, low molecular weight. (**d**) AngII infusion suppressed adipose APN mRNA expression which was not affected by adenoviral APN expression in hyperlipidemic LDLR^−/−^ mice (n = 8/group), *p < 0.05 Student’s t-test (**e**) Immunolocalization of APN in abdominal aorta (red arrows) from AngII-infused AdGFP or AdAPN mice. Mice infused with PBS were used as control (n = 8). Abdominal aortic cross-sections represented in boxes are shown at higher magnification. Scale bar = 50 μm.

To induce chronic and sustained elevation of plasma APN levels, we used a recombinant adenovirus expressing mouse APN (AdAPN) and as control adenovirus expressing green florescent protein (AdGFP) in AngII-infused high-fat fed LDLR^−/− ^11. Administration of AdAPN significantly increased plasma APN levels in AdAPN mice with peak APN levels on day-7 and significantly higher plasma APN levels compared to levels in AdGFP mice were maintained during 8 weeks of the study (Fig. 1a,b). Plasma APN levels measured by ELISA and Western blotting eight weeks after viral injection showed 10-fold elevation in AdAPN mice compared to those in AdGFP mice (Fig. 1a,b). Since APN circulates in high-molecular weight oligomeric forms with major APN bioactivity^17^, we measured their levels^11^. APN expression significantly elevated the plasma levels of high- (HMW) (>30%) and medium-molecular weight (MMW) (>20%) APN oligomers in AdAPN mice compared to the levels (<9%) in AdGFP mice (Fig. 1c). Consistent with our previous report^11^, AngII-infusion supressed APN mRNA levels in white adipose tissue (WAT) and adenoviral APN expression had no impact on endogenous APN levels in WAT (Fig. 1d). Next, we determined whether circulating APN gained access to the arterial wall. Immunostaining of APN in abdominal aorta demonstrated increased APN localization in AdAPN mice compared to that in AdGFP mice (Fig. 1e). Thus, increased APN levels enhanced APN localization in the artery wall^11^. Elevation of APN levels in AdAPN mice had no effect on body weights, plasma cholesterol, triglyceride and glucose levels and blood pressure (Supplementary Table 1).

### Elevated adiponectin levels inhibit AngII-induced advanced AAA development

**Figure 2:**
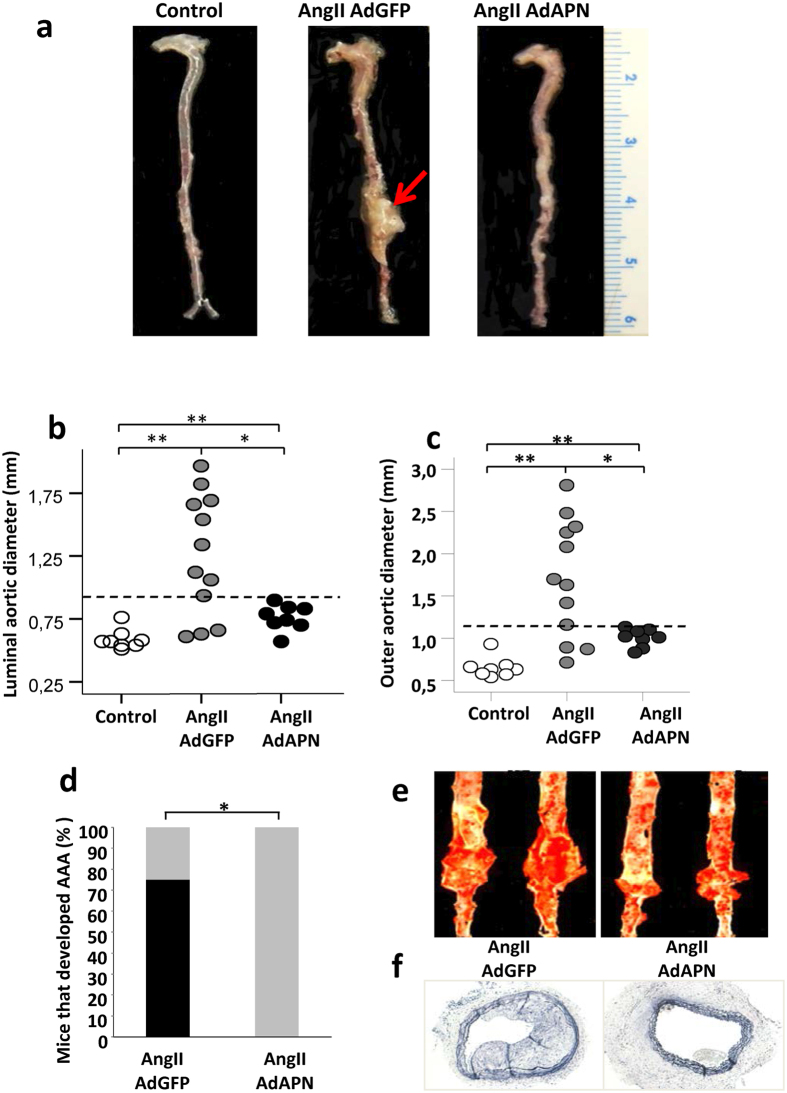
Adiponectin elevation inhibits AngII-induced AAA formation. (**a**) Representative photographs showing macroscopic view of AAA induced by AngII infusion (red arrow). (**b**) Luminal diameter of abdominal aortas. Control n = 8, AdGFP n = 12, AdAPN n = 8, **p < 0.01 vs AdGFP and AdAPN, *p < 0.05 vs AdGFP, ANOVA (**c**) Outer aortic diameter of abdominal aortas. Control n = 8, AdGFP n = 12, AdAPN n = 8 **p < 0.01 vs AGFP and AdAPN, *p < 0.05 vs AdGFP, ANOVA. (**d**) Percent of AngII-infused LDLR^−/−^ mice injected AdAPN or Ad-GFP that developed advanced AAA. *p < 0.05 vs AdGFP (**e**) Sudan IV-stained *en face* aortic preparations showing abdominal atherosclerotic lesions and aneurysm. (**f**) Representative cross-sections of abdominal aortas from AngIIAdGFP and AngIIAdAPN showing atherosclerosis (magnification 10X).

We investigated whether enhanced APN expression provides therapeutic benefit against AngII-induced AAA by injecting AdGFP or AdAPN into AngII-infused hyperlipidemic LDLR^−/− ^11,18. The rationale for using prolonged AngII infusion for 8 weeks instead of 4 weeks is to determine whether APN prevents not only the initial AAA devlopment but also its progression and expansion into advanced AAA. AngII infusion for 8 weeks induced large and advanced AAA development in AdGFP mice (Fig. 2a). Remarkably, APN expression largely inhibited AAA development in AdAPN mice (Fig. 2a). The definition of an aneurysm was set at a 1.5-fold enlargement of the lumen and outer diameter. When extracting mouse aorta for aortic outer diameter measurements, the biggest part of the aorta was taken for this purpose. Using a threshold of aortic luminal diameter for aneurysm set at 0.77 mm (50% increase) from baseline PBS-infused control mice (0.51 ± 0.05 mm), AdAPN mice (0.53 ± 0.12 mm) demonstrated a 40% reduction in the aortic luminal diameter compared to AdGFP mice (0.88 ± 0.45 mm, p < 0.05) (Fig. 2b). AdAPN mice (1.01 ± 0.1 mm) exhibited significantly reduced (41% reduction) aortic outer diameter compared to that of AdGPF mice (1.69 ± 0.7 mm, p < 0.05) (Fig. 2c). Thrombus formation in the aortic wall was observed in 15% of AdGFP mice but not in AdAPN mice. No aortic rupture was observed in both the AngII-infused AdGFP and AdAPN mice. In AdGFP mice, increased arterial wall thickness was associated with aortic dissection, intraluminal thrombus, interface fluid, atherosclerosis and connective tissue deposition, all hallmarks of AAA. In contrast, AdAPN mice exhibited a more preserved and stable artery wall with reduced atherosclerosis. Aneurysm development was found in 75% of AdGFP mice compared to none in AdAPN mice. Thus, APN expression significantly prevented AngII-induced AAA. Furthermore, consistent with our previous report that APN inhibited atherosclerosis in the entire aorta in this model^11^, we found reduced abdominal atherosclerotic lesions in AdAPN mice compared to those in AdGFP mice (Fig. 2e,f).

### Adiponectin inhibits AngII-induced elastin degradation, inflammatory cell infiltration and vascular smooth muscle cell degeneration in the abdominal aorta

**Figure 3:**
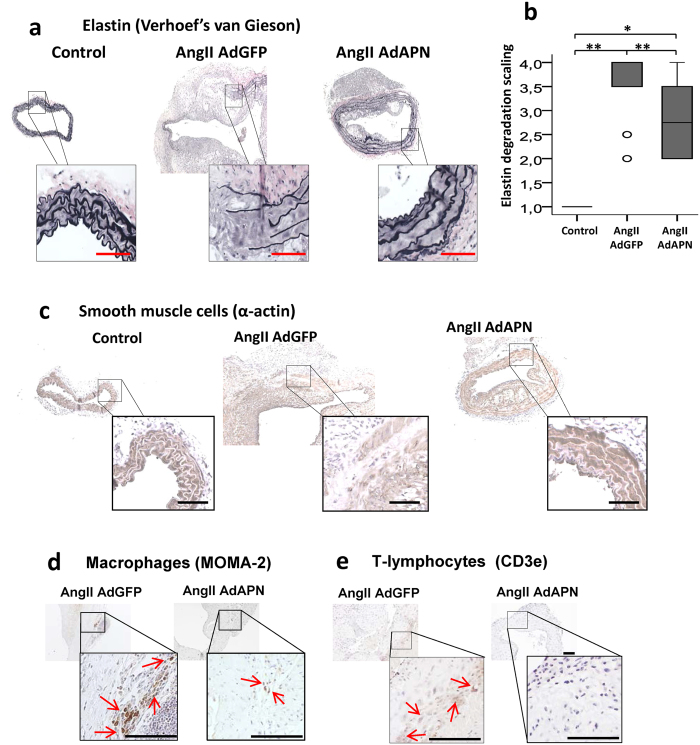
Adiponectin expression inhibited AngII-induced elastin degradation, infiltration of inflammatory cells and preserved vascular smooth muscle cells in the abdominal aorta of hyperlipidemic LDLR^−/−^ mice. (**a**) Elastin staining using Verhoeff’s van Geison stain in representative abdominal aorta cross-sections from control (PBS) and AngII-infused and AdGFP or AdAPN injected mice after 8 weeks. (**b**) Quantification of elastin degradation in abdominal aorta of control (PBS-infused, n = 8) and AngII-infused and AdGFP (n = 12) or AdAPN (n = 8) injected mice after 8 weeks. Results are presented in box plot using median (line) with the 25th and 75th percentiles. T-bars indicate outliers 1.5 times the box height and the circles 1.5 times the interquartile range. **p < 0.01 vs AdGFP and Control, *p < 0.05 vs AdAPN, ANOVA. (**c**) Representative immunostained images of vascular smooth muscle cells (SMC22α) localization in the abdominal aortic cross-sections from control (PBS-infused) and AngII-infused and AdGFP or AdAPN injected mice after 8 weeks. Immunolocatization of macrophages (MOMA2) (**d**) and T-lymphocytes (CD3e) (**e**) in abdominal aorta from AngII-infused and AdGFP or AdAPN injected mice after 8 weeks. Infiltration of inflammatory cells is detected only in AngII-infused mice and not in PBS-infused mice. Red arrows represent macrophage-rich (**d**) and T-lymphocyte-positive (**e**) areas. Areas of abdominal aortic cross-sections (**a,c,d,e**) represented in boxes are shown at higher magnification. Scale bar = 50 μm.

Chronic inflammation due to the infiltration of macrophages, T-lymphocytes and mast cells leads to degeneration of medial VSMC and destruction of collagen and elastin, which maintain aortic wall integrity^19^,^20^. We investigated whether APN protects from AngII-induced elastin fragmentation in the aneurysmal wall. Verhoeff´s staining and elastin scoring of abdominal aortic sections demonstrated extensive elastin fragmentation in all AdGFP mice (Fig. 3a). Remarkably, APN expression substantially reduced elastin fragmentation in AdAPN mice (Fig. 3a,b). Since medial VSMC degeneration is a key feature of AAA^4^,^21^, we examined the effect of APN on AngII-induced medial degeneration by immunostaining of VSMC (SM22α) in the abdominal aorta. Our results showed extensive medial damage and reduced VSMC staining in AdGFP mice (Fig. 3c), in contrast to intact media and well-preserved VSMC in AdAPN mice (Fig. 3c). Thus, APN inhibited AngII-induced aortic medial degeneration. Since AngII induces transmural infiltration of macrophages and T-lymphocytes^3^, we determined if APN impacts on inflammatory cell infiltration of aortic wall. Immunolocalization of macrophages (MOMA2) and T-lymphocytes (CD3e) in abdominal aortic sections revealed substantially reduced macrophage (Fig. 3d) and T-lymphocyte staining (Fig. 3e) in AdAPN mice compared to that in AdGFP mice. These results provide strong evidence that APN inhibited AngII-induced accumulation of macrophages and T-lymphocytes in the abdominal aorta. This supports our previous report that APN inhibits AngII-mediated vascular inflammation^11^ and APN is protective adipokine to target AAA. Our current studies elucidate therapeutic potential of APN intervention on advanced AAA development, under AngII-suppression of endogenous APN expression in WAT.

### Adiponectin regulates AngII-mediated inflammatory and extracellular matrix gene expression in the abdominal aortic wall

**Figure 4:**
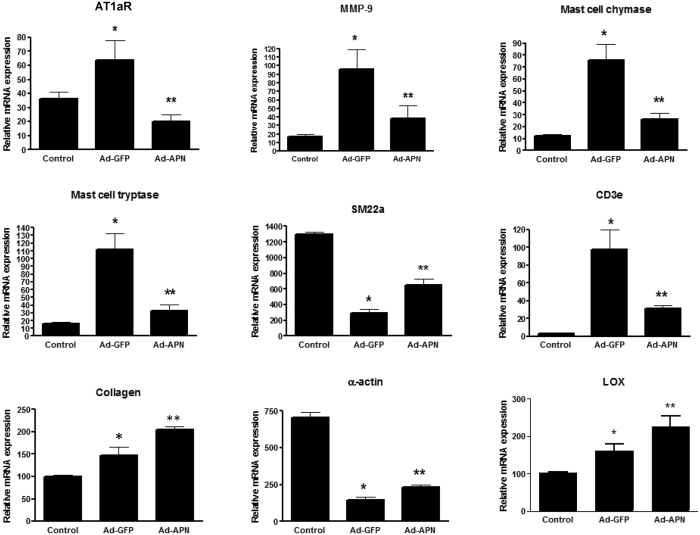
Adiponectin regulates AngII-mediated inflammatory and extracellular matrix gene expression in the aneurysmal wall. Gene expression analysis of angiotensin type 1a receptor (AT1aR), MMP9, mast cell chymase, mast cell tryptase, smooth muscle cell marker (SM22α), T-lymphocyte marker (CD3e), collagen, α-actin and lysyl oxidase (LOX) in abdominal aorta of control (PBS-infused) (n = 8) and AngII-infused AdGFP (n = 12) or AdAPN (n = 8)-injected mice after 8 weeks after AngII infusion. AT1aR analyzed with n = 5/group. RNA samples from abdominal aorta were analyzed by QRT-PCR and normalized to TBP. *p < 0.05 vs Control, **p < 0.01 vs AdGFP and Control, ANOVA.

To further explore potential mechanisms by which APN inhibited aneurysm development, we performed gene expression analyses of abdominal aortas. AngII actions contributing to aortic inflammation and damage are mediated by the AT1aR^22^. We previously reported that APN inhibits aortic AT1aR in the AngII-induced LDLR^−/−^ model^11^. Here, we show a significant attenuation of AT1aR by APN in the abdominal aorta of AdAPN mice (Fig. 4). Gene expression analyses showed a significant reduction of VSMC marker (SM22α and α-actin) mRNA levels in both AdGFP and AdAPN mice compared to control mice (Fig. 4). However, in AdAPN mice both SM22α and α-actin levels in the abdominal aorta were significantly increased compared to the levels in AdGFP mice (Fig. 4). Mast cells play a key role AAA pathogenesis and mast cell proteases, chymase and tryptase, promote vascular inflammation, SMC apoptosis and elastinolysis^23^,^24^,^25^. We found that both mast cell chymase and tryptase mRNA levels were significantly increased in AngII-infused AdGFP mice (Fig. 4). Interestingly, APN inhibited aortic mast cell chymase and tryptase levels in AdAPN mice (Fig. 4). Consistent with reduced T-lymphocyte staining, CD3e expression is reduced in the abdominal aorta (Fig. 4). Next, we detemined the effect of APN on matrix degrading proteases and collagen production in aortic wall^24^. Our results showed a significant suppression of matrix metalloproteinase-9 (MMP-9)^26^, and increase in collagen expression in AdAPN mice (Fig. 4). Importantly, we sought potential mechanisms contributing to APN inhibition of elastin fragmentation in AdAPN mice. Gene expresson analysis revealed that in AdAPN mice APN expression upregulated LOX expression (Fig. 4) which promotes elastin cross-linking and aortic wall integrity^27^,^28^. The increased expression of collagen and LOX in the abdominal aorta of AdGFP mice appears to be related to compensatory production of collagen and elastin due to enhanced degradation induced by AngII. Overall, these results demonstrate that increased APN expression attenuated AngII-mediated inflammatory signaling and proteinase levels as well as induced LOX and collagen expression which contribute to preservation of arterial wall intergrity.

### Adiponectin expression improves systemic inflammatory cytokine/chemokine profile

**Figure 5:**
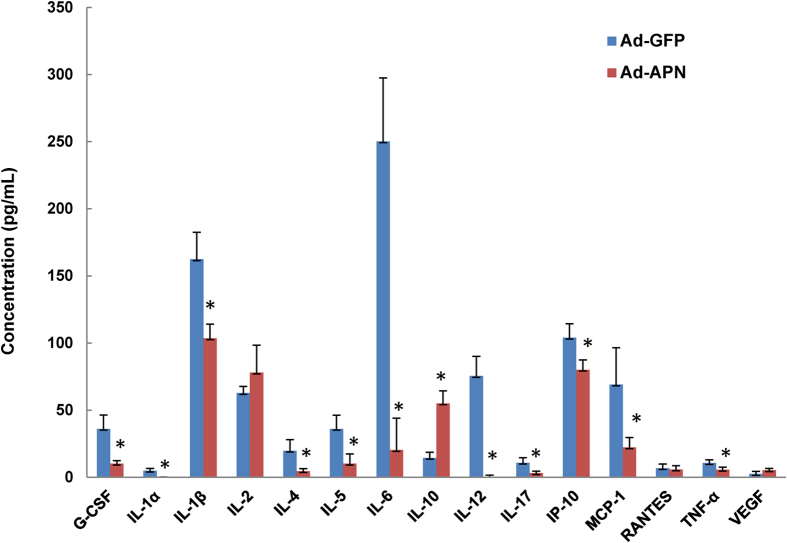
Adiponectin expression improves systemic inflammatory cytokine/chemkine profile. Circulating cytokine/chemokine and growth factor levels in AngII-infused hyperlipidemic LDLR^−/−^ mice, 8 weeks after injection of AdGFP or AdAPN. Plasma samples pooled from individual mice from AdGFP (n = 9) and AdAPN (n = 8) group were analyzed by multiplex ELISA immunoassay. *p < 0.05 vs AdGFP, Student’s t-test.

Circulating inflammatory cytokines are elevated in AAA^3^,^20^,^29^,^30^. We determined the effect of elevated APN levels on systemic cytokine, chemokine and growth factor profile by multiplex ELISA analysis of plasma from AdAPN and AdGFP mice (Fig. 5). Notably, in AdAPN mice, circulating levels of G-CSF, IL-1α, IL-1β, IL-4, IL-5, IL-6, IL-12, IL-17, IP-10, MCP-1 and TNF-α were markedly reduced and the levels of IL-10 were significantly increased compared to those in AdGFP mice (Fig. 5). Thus, APN improved systemic inflammatory profile by reducing inflammatory mediators and increasing anti-inflammatory IL-10 which is consistent with reduced aortic inflammation and inhibition of AAA. Precise mechanisms by which APN induces local anti-inflammatory response remains to be established.

### Adiponectin inhibits perivascular and visceral adipose inflammation

**Figure 6:**
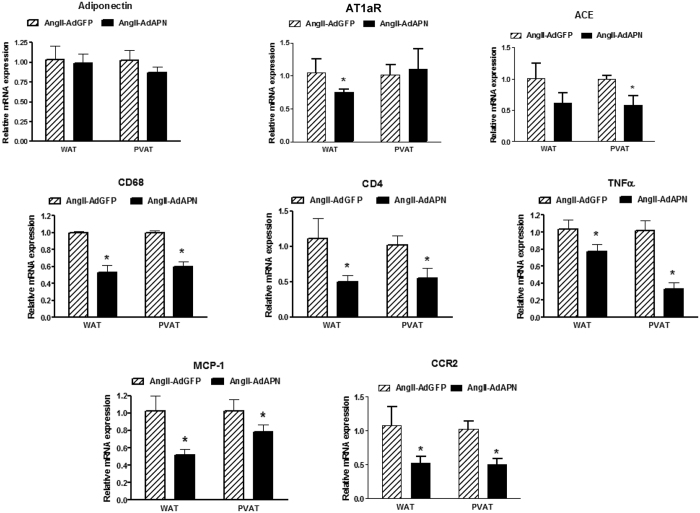
Adiponectin inhibits perivascular (abdominal) and visceral adipose inflammatory cell infiltration, renin-angiotensin system component and inflammatory cytokine/chemokine expression. Gene expression analysis of adiponectin (APN), angiotensin type 1a receptor (AT1aR), angiotensin-converting enzyme (ACE), macrophage marker (CD68), T-cell marker (CD4), Tumor necrosis factor alpha (TNF-α), monocyte chemoattractant protein-1 (MCP-1) and chemokine receptor 2 (CCR2) in perivascular adipose tissue (surrounding abdominal aorta) of AngII-infused mice 8 weeks after injection of AdGFP or AdAPN (n = 8/group). RNA samples from abdominal perivascular adipose tissue aorta were analyzed by QRT-PCR and normalized to GAPDH. *p < 0.05 vs AdGFP, Student’s t-test.

**Figure 7:**
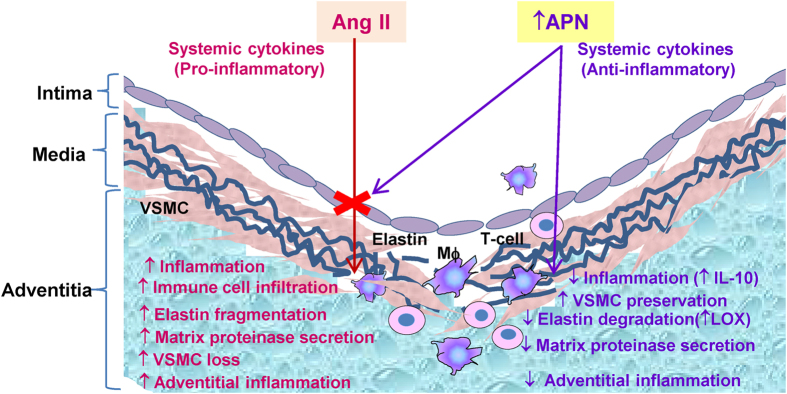
Schematic showing potential mechanisms by which APN inhibits AngII-induced advanced AAA development. This study shows that the protective APN actions are mediated by multiple mechanisms including inhibition of the RAS components, inflammatory cell infiltration, vascular SMC degeneration and elastic fragmentation in the aneurysmal wall. In addition, APN suppressed perivascular and visceral adipose inflammation. (Mφ, macrophage, ↑ increase, ↓ decrease).

Several studies have linked obesity to AAA and both perivascular and visceral adipose tissue inflammation contribute to AAA pathogenesis^30^,^31^. We investigated whether increased APN levels regulate inflammatory status of perivascular adipose surrounding abdominal aorta (PVAT) and visceral white adipose tissue (WAT). In hyperlipidemic LDLR^−/−^ model, we found that endogenous APN is predominently expressed in WAT rather than in PVAT surrounding abdominal aorta (Supplementary Fig. 1). As in WAT^11^, adenoviral APN did not affect endogenous APN levels in PVAT (Fig. 6). Interestingly, we found that elevated APN inhibited mRNA levels of angiotensin converting enzyme (ACE) in PVAT and AT1aR in WAT in AdAPN mice (Fig. 6). Furthermore, increased APN levels inhibited both macrophage (CD68) and lymphocyte (CD4) marker levels in PVAT and WAT in AdAPN mice compared to those in AdGFP mice (Fig. 6). This was accompanied by significant inhibition of TNF-α, MCP-1 and CCR2, the key players in adipose macrophage recruitment and inflammation. These findings suggest direct and local anti-inflammatory and inhibitory actions of APN on adipose RAS which may have contributed to inhibition of AAA (Fig. 7). Nevertheless, whether these APN actions are direct or a consequence of complex pathological changes associated with AAA progression remain to be established in future studies.

## Discussion

In the present study, we investigated the therapeutic role of increasing plasma APN levels on AngII-induced advanced AAA development in a well-established preclinical model. There has been increased interest in the role of obesity and adipose-derived factors in the control of vascular function and remodeling. Growing evidence suggests that chronic inflammation in obesity-related disorders due to increased inflammatory adipokine production and the RAS activation contributes to cardiovascular complications. Although inflammatory adipokines, leptin and resistin are linked to promoting AAA their pathogenic role in AAA remains to be established^9^,^10^. Our study is focused on APN, an adipokine with profound anti-inflammatory and anti-athergenic properties. We have previously established that APN expresson to increase plasma APN levels effectively inhibits AngII-mediated vascular inflammation and accelerated atherosclerosis^11^. Consistent with these studies, complete knockout of APN in apolipoprotein E-deficient mice has been reported to enhance AngII-induced inflammation and early AAA^8^. In this present study, we established the therapeutic role of increasing plasma APN levels to prevent AngII-induced advanced AAA using the hyperlipidemic LDLR^−/−^ mice. In this model, continuous AngII infusion for 8 weeks leads to progressive AAA expansion and aortic remodeling with complex pathological features consistent with advanced AAA^32^. The rationale for using prolonged AngII infusion for 8 weeks instead of 4 weeks is to determine whether APN prevents not only initiation of AAA (4 weeks) but also inhibit the later events of progression and expansion into advanced AAA (8 weeks). Using this advanced AAA model, we clearly demonstrated the theraputic benefit of increasing plasma APN levels to prevent AngII-induced AAA. Furthermore, our study provides strong evidence that the protective APN actions are mediated through multiple mechanisms including inhibition of aortic and perivascular inflammatory cell infitration, suppression of inflammatory mediators and the RAS components to inhibit systemic, aortic wall and adipose (perivascular/visceral) inflammation. This study also highlights the protective role of APN to prevent VSMC degeneration and elastin fragmentation in the aneurysmal wall (Fig. 7). These findings strongly support the therapeutic role of APN in preventing AngII-induced advanced AAA development.

In humans, AAA patients frequently have underlying atherosclerosis and it is unclear whether atherosclerosis plays a causal role or shares common risk factors with AAA. Atherosclerosis develops in parallel rather than AAA formation caused by atherosclerosis^6^. Our results have shown that APN inhibition of AAA is also accompanied by marked reduction of atherosclerosis in the aneurysmal wall. This is consistent with our previous report in the same model that APN inhibits atherosclerosis throughout the aorta including the aortic root. Since vascular inflammation is a major factor in the pathogenesis of both AAA and atherosclerosis and a key mechanism by which APN protects against AAA is through anti-inflammatory effects in the aortic wall. It is well-established that AngII induces atherosclerosis and AAA development through stimulation of AT1aR^22^ without affecting the blood pressure^33^. The key role of AT1aR in AAA development is underscored by the evidence that AT1aR blockers (ARBs) inhibit AngII-induced AAA development in rodent models^34^,^35^ and that AT1aR polymorphisms are associated with AAA in humans^36^. We previously reported that APN inhibits AngII-induced AT1aR expression whereas it increased AT2R expression in the aortic wall^11^. Interestingly, in the current study, we demonstrated that APN markedly inhibited AngII-induced AT1aR expression in the abdominal aorta without a significant effect on blood pressure. Thus, suppression of AT1aR in the aneurysmal wall is one potential mechanism by which APN protects against AngII-mediated aortic inflammation and damage.

The transmural inflammation due to the infiltration of macrophages, lymphocytes and mast cells leads to induction of inflammatory cytokines, loss of medial VSMC and destruction of elastin and collagen which contribute to AAA development^37^. Loss of VSMC impairs synthesis of both collagen and elastin, key ECM components which provide tensile strength and elasticity to the aortic wall. Our study demonstrated that increased APN levels largely prevented AngII-induced elastin degradation and preserved the aortic wall integrity. In addition, other potential mechanisms contributing to this effect include suppression of the mast cell chymase and MMP-9 expression that contribute to the weakening of the arterial wall and aneurysmal dilation. In this study, we also identified LOX as an APN target contributing to the APN inhibition of elastin fragmentation in aneurysmal wall. LOX encodes a key amine oxidase that cross-links elastin and collagen fibers to maintain artery wall integrity. Evidence suggests that LOX is critical in AAA development and increased LOX expression prevents AAA^27^,^28^,^38^. Our results showed that APN increased aortic LOX expression consistent with prevention of elastin degradation and AAA development in AdAPN mice. Furthermore, there is evidence that LOX is anti-inflammatory by suppressing MCP-1^39^. Thus, increased LOX expression by APN may have contributed to attenuation of inflammation as well as ECM stability. In addition, we found that elevated APN levels significantly increased collagen expression in the aortic wall of AdAPN mice. This suggests a potential compensatory mechanism in response to increased protease activity in the aneurysmal wall. Collagen synthesis increases during the early stages of aneurysm formation, as a repair process, and in the later stages, collagen degradation exceeds the synthesis^40^. The increased collagen and LOX expression in AdGFP mice compared to PBS-infused controls appears to be a compensatory response due to enhanced degradation of ECM induced by AngII. Although increased collagen content is thought to play a protective role in maintaining arterial wall integrity in the context of AAA, excess collagen accumulation can contribute to arterial wall stiffness and dysfunction^41^. In this study, we demonstrated that APN inhibited AngII-induced medial VSMC degeneration as shown by increased VSMC immunostaining and expression of VSMC markers (SM22a and α-actin) in the aneurysmal wall. These findings support the protective role of APN to inhibit AngII-induced medial degeneration and to preserve ECM production in the aortic wall. Other mechanism contributing to VSMC degeneration includes mast cell chymase which induces apoptosis by disrupting NF-κB mediated survival signaling^42^. We found that increased APN levels suppressed mast cell chymase expression in the aneurysmal wall, which, in part, may have contributed to the inhibition of elastin degradation and loss of VSMC in the aortic wall.

The mast cells are one of the major inflammatory cells present in AAA^25^,^43^. Activated mast cells produce a wide range of inflammatory cytokines/chemokines and mast cell-specific proteases, chymase and tryptase which induce MMP activation and AAA progression^44^. Mast cell chymase and tryptase are increased in experimental and human AAA^45^. The importance of mast cell in AAA is highlighted in studies in rodent models demonstrating that the absence of mast cells or inhibition of mast cell proteases protects from AAA. In the present study, we found a substantial increase in the mast cell chymase and tryptase expression in the abdominal aorta after 8 weeks of AngII infusion. Remarkably, APN inhibited mast cell chymase and tryptase in the aortic wall. Mast cells secrete various inflammatory mediators capable of activating T lymphocytes through TNF-α and macrophages. In human AAAs, several studies have identified a Th2 predominant immune response with Th2-associated cytokines (IL-4, and IL-10), while no or little expression of Th1-associated cytokines (IL-2, IL-12, IL-15 and IFN-γ)^46^. In contrast to AAA, atherosclerotic lesions contain predominantly Th1-associated cytokines^47^. The CD4+ T lymphocytes and INF-γ play an important pathogenic role in AAA. Deficiency of either CD4 or INF-γ prevents CaCl_2_-induced aneurysm. However, the aneurysm could be reconstituted in CD4^−/−^ mice with INF-γ injections, suggesting an essential role of T lymphocytes in AAA formation^48^. In addition, Th2 produced IL-10 promotes the death of Th1 cells and activation of anti-inflammatory M2 macrophages. Production of IFN-γ by Th1 cells can in turn activate macrophages and stimulate production of inflammatory cytokines, such as those produced by the classical macrophages (M1 macrophages), e.g. IL-12 and TNF-α involved in tissue injury. In contrast to the inflammatory M1 macrophages, M2 macrophages have an anti-inflammatory role and regulates tissue repair through production of anti-inflammatory IL-10 and transforming growth factor-β1 (TGF-β1)^16^,^49^. In this study, we found that APN substantially reduced both macrophage and T-lymphocyte infiltration accompanied by the suppression of mast cell proteases, tryptase and chymase, in the abdominal aorta of AdAPN mice. In the context of cytokine expression in the aortic wall after 8 weeks of AngII infusion, we previously reported that aortic macrophages in AdGFP mice are predominantly M1 and that in AdAPN mice, APN induced IL-10 expression and suppressed inflammatory cytokines^11^. Furthermore, there is *in vitro* evidence that APN induces IL-10 expression in macrophages^16^. Taken together, these studies suggest the predominance of anti-inflammatory Th2 cells and M2 macrophages in the aorta of AdAPN mice. Supporting this notion, our analysis of systemic plasma cytokine/chemokine profile revealed marked reduction of inflammatory cytokine levels (e.g., IL-6, IL-12, IL-17) and elevation of IL-10 levels in AdAPN mice.

Obesity is a major risk factor for cardiovascular disease^12^. There is evidence linking obesity to AAA development and that increased infiltration of macrophages contributes to both PVAT and WAT inflammation^30^,^31^. Consistent with this concept, we demonstrated that APN levels regulate the inflammatory status of both PVAT and WAT. Elevated APN levels inhibited macrophage and T-lymphocyte infiltration into PVAT and WAT by inhibiting TNF-α, MCP-1 and CCR2 as well as components of local RAS. These results provide first evidence that increased APN levels exert local protective actions in PVAT and WAT to inhibit adipose inflammation and AAA development. These findings underscore the potentially important role of APN in the control of of inflammatory and local RAS components in adipose inflammation. In the context of RAS, it is noteworthy that RAS inhibition by AT1aR blockers (ARBs) and ACE inhibitors is often accompanied by increased plasma APN levels^50^. Given the multiple protective actions of APN in AngII-induced AAA, it is possible that some of the vascular protection by the RAS inhibitors may be, in part, due to elevation of APN levels. It is conceivable that secondary factors associated with the presence of AAA in AdGFP mice, which were absent in AdAPN mice, can contribute to advanced AAA development. Thus, future studies to determine APN actions on preexisting AAA and regression will be of considerable interest.

In conclusion, our studies demonstrated for the first time that increasing plasma APN levels provided therapeutic benefit preventing the development of AngII-induced advanced AAA in a well-established preclinical model. The protective APN actions are mediated through multiple mechanisms including inhibition of the RAS components, perivascular and aortic inflammatory cell infiltration and suppression systemic, aortic wall and perivascular adipose inflammation (Fig. 7). Furthermore, this study highlights the protective role of APN to prevent VSMC degeneration and elastin fragmentation by suppressing mast cell proteinases and inducing LOX to stabilize the aortic wall (Fig. 7). Further studies are warranted to investigate various therapeutic strategies promoting APN levels and elucidate integrated APN targets to prevent advanced AAA development as well as their role in potential regression of established AAA.

## Methods

### Mouse model of AngII-induced hyperlipidemic aortic abdominal aneurysm

In this model, infusion of AngII for 8 weeks in male LDLR^−/−^ mice leads to development of advanced AAA in up to 80% of mice. In this model, continuous AngII infusion for 8 weeks leads to progressive AAA expansion and aortic remodeling with complex pathological features consistent with advanced AAA^32^. The rationale for using prolonged AngII infusion is to determine whether APN prevents not only initiation of AAA (4 weeks) but also provides protection beyond initiaton by preventing the later events of progression and expansion into advanced AAA (8 weeks). Male LDLR^−/−^ mice (8–10 weeks old) were purchased from Jacksons Laboratory (Bar Harbor, ME, USA). Mice were fed a high-fat diet (Research diets, New Brunswick, NJ, USA, Western diet D12079B, protein, 17kcal%; carbohydrate, 43 kcal%; fat, 41kcal%) and water was allowed *ad libitum* throughout the study. To induce AAA, mice were anesthetized by inhalation of isofluorane (0.5–1.5% vapor) and mini-osmotic pumps (Model 1004, Alzet, CA, USA) were implanted subcutaneously into the right flank on each mouse, releasing AngII (1.5 μg/kg/min, Sigma Aldrich, St Louis, USA). At the implantation site, analgesics bupivicane (2.5 mg/kg sc) and carpofen (5.0 mg/kg, sc) were applied pre- and post-implantation, respectively. After 4 weeks of AngII infusion, new replacement 4-week mini-osmotic pumps were implanted to continue AngII infusion for a total of 8 weeks. A group of mice were infused with phosphate-buffered saline (PBS) as controls (n = 8). To induce APN expression, a recombinant adenoviral vector encoding full-length mouse adiponectin (AdAPN, 2 × 10^8^ pfu) was injected intravenously via retroorbital plexus into mice (n = 8). Animals were fed high-fat diet at the same time as AngII infusion and the adenoviral vectors were injected within 24 hours of AngII infusion pump implantation. A recombinant adenovirus expressing green fluorescence protein (AdGFP, 2 × 10^8^ pfu) was used (AdGFP, n = 12) as a control vector. Blood pressure was monitored weekly by the tail-cuff method using BP-200 Visitech System Inc. (Apex, NC, USA). After 56 days, mice were sacrificed and whole blood was drawn by jugular vein puncture and the aorta was removed and fixed in 4% formalin for histological analyses and RNAlater (Ambion, Austin, TX, USA) for gene expression analyses^11^. Aortas dissected and pinned out were stained with Sudan IV. For additional groups of mice, perivascular tissue surrounding the abdominal aorta (below the diaphragm) and white adipose tissue from visceral cavity were dissected out and stored at −80 °C for RNA isolation. The suprarenal aortic diameter was measured as the outer and luminal diameter and the definition of an aneurysm was set as a 1.5 fold enlargement of the aortic wall. To measure aortic outer diameter, the biggest part of the aorta was taken for this purpose. Animal studies performed conform to NIH guidelines and were approved by the Animal Research Committee, University of California, Los Angeles, CA.

### Recombinant adenoviral vector construction

To generate pAxCAwt-mouse APN, cDNA encoding mouse APN was inserted in the pAxCAwT plasmid (TAKARA Biomedical, Shiga, Japan). The plasmid containing mouse APN cDNA was driven under the control of CMW enhancer, chicken β-actin promoter and an untranslated region of rabbit β-globin^11^.

### Gene expression analysis

Suprarenal aortas and perivascular and white adipose tissue were homogenized with Tissue Lyser using safe-lock tubes with metal bead and trizol-chloroform. Total aortic RNA was isolated with RNeasy Mini kit (Qiagen, Hilden, Germany) and reverse transcribed with random primers and Superscript III (Invitrogen, Carlsbad, CA, USA). cDNA (1.0 ng) was amplified by RT-PCR with TaqMan Universal PCR Mastermix (Applied Biosystems, Foster City, CA, USA) using 7500 Fast Real-time PCR Sequence Detector (Applied Biosystems)^11^,^51^. Samples were run in duplicate and semi-quantified against a standard curve. All probes were obtained from Applied Biosystems and results were normalized to mouse TBP.

### Determination of plasma APN, lipids, glucose and APN oligomeric forms by Western blot

Plasma samples collected from mice after 5 hr fasting were analyzed for cholesterol, triglycerides by enzymatic methods (Wako Chemicals USA Inc, Richmond, VA, USA). Blood glucose levels were measured by one-touch glucose monitoring system (WAKO)^11^,^51^. Plasma APN levels were measured by Adiponectin ELISA kit (Otsuka Pharmaceuticals Inc, Rockville, MD, USA). Plasma APN oligomer form levels were determined by Western blot as previously described^11^,^17^.

### Elastin and collagen staining

Paraffin embedded abdominal aortic sections (5 μm) were stained with Verhoeff´s hematoxylin for 1 h, differentiated in 2% ferric chloride for 2 min and counterstained with Van Gieson solution for 5 min to identify elastic fibers in the aorta tissue. Quantification of elastin was performed in a blinded fashion by two investigators. A scoring system from 1–4 was used where score 1 is defined as intact elastin, 2 as low elastin degradation, 3 as intermediate elastin degradation and 4 as high elastin degradation. For collagen, aortic sections (5 μm) were stained with Sirius Red (Sigma Aldrich) for 1 h and washed twice with 1% acetic acid for 10 min. Measurements were made using the LeicaQWin image analysis software (Leica, Wetzlar, Germany).

### Immunohistochemical staining

Paraffin embedded mouse abdominal aortas were sectioned (5 μm), rehydrated and treated with 1× Diva antigen retrieval solution (Biocare Medical, Concord, CA, USA) to unmask the antigen. Endogenous peroxidase activity was quenched by treatment with 3% hydrogen peroxidase for 5 min followed by incubation in 5% normal goat serum (Vector Laboratories, Burlingame, USA). Sections were then incubated with rat monoclonal MOMA (AbD Serotec, Oxford, UK), α-actin (Sigma Aldrich) and mouse polyclonal SM22α (Abcam, Cambridge, UK) and CD3 (Abcam) antibodies at 4 °C overnight followed by corresponding secondary biotinylated goat anti-rabbit, goat anti-rat or goat anti-mouse (Vector Laboratories). Isotypic IgG in the same concentration served as negative control (Santa Cruz Biotechnologies). Avidin-Biotin peroxidase complex (Dako, Glostrup, Denmark) were added, followed by visualization with 3,3´- diaminobenzidine tetrahydrochloride (Dako). All sections were counterstained with Mayers hematoxylin (Histolab Products, Gothenburg, Sweden).

### Determination of plasma mediators by multiplex ELISA immunoassay

Blood samples were collected in tubes containing EDTA and then centrifuged to separate plasma. Analysis were performed with pooled terminal plasma from Ad-GFP mice (n = 9) and Ad- APN mice (n = 8) at 56 days of treatment. Systemic cytokines/chemokines/growth factors were measured using a high-sensitivity multiplex ELISA immunoassay (Biorad-Bioplex) including granulocyte colony-stimulating factor, IL-α, IL-1β, IL-2, IL-4, IL-5, IL-6, IL-10, IL-12, IL-17, IP-10, MCP-1, RANTES, TNF-α and VEGF.

### Statistical Analysis

All data were expressed as mean ± standard deviation. The statistical significance was analyzed using GraphPad Prism software. Statistical significance of quantitative data was determined using Student´s t-test and ANOVA (Newman-Keuls post-test) or non-parametric (Mann-Whitney) tests as appropriate depending on normal distribution. Statistical differences for all comparisons were considered significant at p < 0.05.

## Additional Information

**How to cite this article**: Wågsäter, D. *et al*. Elevated Adiponectin Levels Suppress Perivascular and Aortic Inflammation and Prevent AngII-induced Advanced Abdominal Aortic Aneurysms. *Sci. Rep.* **6**, 31414; doi: 10.1038/srep31414 (2016).

## Supplementary Material

Supplementary Information
