# Supplementary material for: Elevated Adiponectin Levels Suppress Perivascular and Aortic Inflammation and Prevent AngII-induced Advanced Abdominal Aortic Aneurysms
Source: Sci Rep. 2016 Sep 23;6:31414. doi: 10.1038/srep31414 (PMC5034224; doi:10.1038/srep31414)
Supplement: Supplementary Information [file srep31414-s1.pdf]

Elevated Adiponectin Levels Suppress Perivascular and Aortic Inflammation and Prevent AngII-induced Advanced Abdominal Aortic Aneurysms

Dick Wågsäter, Emina Vorkapic, Caroline M.W. van Stijn, Jason Kim, Aldons J Lusis, Per Eriksson and Rajendra K. Tangirala

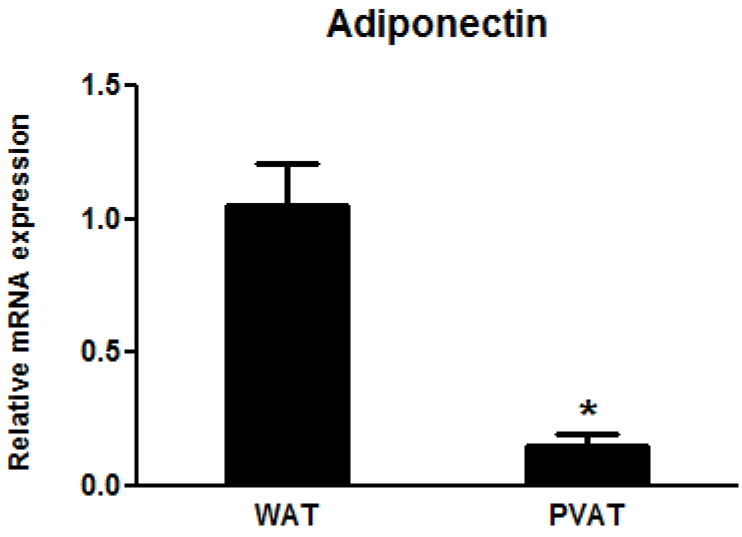

Supplementary Figure 1. Adiponectin mRNA expression in white adipose tissue (WAT) and perivascular adipose tissue (PVAT) surrounding the abdominal aorta in hyperlipidemic LDLR$^{-/-}$ mice. Adiponectin expression was measured by QRT-PCR and normalized to GAPDH. *****$p<0.001$ Student's t-test (n=5/group).

Supplementary Table 1

Effect of adiponectin expression on AngII-infused high-fat fed LDLR$^{-/-}$ mice after 8 weeks

	**PBS**	**AngII-AdGFP**	**AngII-AdAPN**
Body weight (g)	31.4 ± 3.1	20.9 ± 1.6*	20.0 ± 1.4*
Adipose tissue weight (g)	1.18 ± 0.7	0.27 ± 1.8*	0.24 ± 0.9*
Blood pressure (mmHg)	127.5 ± 8.1	170.0 ± 12.5*	163.7 ± 24.0*
Total cholesterol (mg/dl)	648 ± 58.0	609.7 ± 79.6	681.2 ± 34.8
Triglycerides (mg/dl)	70.5 ± 17.4	152.7 ± 76.0*	136.6 ± 38.0*
Glucose (mg/dl)	302.0 ± 72.0	270.0 ± 48.4	285.0 ± 22.7

* $p>0.05$ vs PBS
